# Prescription trends in Japanese advanced Parkinson’s disease patients with non-motor symptoms: J-FIRST

**DOI:** 10.1371/journal.pone.0309297

**Published:** 2024-10-23

**Authors:** Masahiro Nomoto, Yoshio Tsuboi, Kenichi Kashihara, Shih-Wei Chiu, Tetsuya Maeda, Hidemoto Saiki, Hirohisa Watanabe, Yasushi Shimo, Nobutaka Hattori, Takuhiro Yamaguchi, on behalf of the J-FIRST Investigators

**Affiliations:** 1 Department of Neurology and Clinical Pharmacology, Ehime University Graduate School of Medicine, Ehime, Japan; 2 Department of Neurology, Faculty of Medicine, Fukuoka University, Fukuoka, Japan; 3 Department of Neurology, Okayama Kyokuto Hospital, Okayama, Japan; 4 Division of Biostatistics, Tohoku University Graduate School of Medicine, Miyagi, Japan; 5 Department of Neurology, Research Institute for Brain and Blood Vessels-Akita, Akita, Japan; 6 Department of Neurology, Kitano Hospital, The Tazuke Kofukai Medical Research Institute, Osaka, Japan; 7 Brain and Mind Research Center, Nagoya University, Aichi, Japan; 8 Department of Neurology, Juntendo University School of Medicine, Tokyo, Japan; University of Catania Department of Surgical and Medical Sciences Advanced Technologies GF Ingrassia: Universita degli Studi di Catania Dipartimento di Scienze Mediche Chirurgiche e Tecnologie Avanzate GF Ingrassia, ITALY

## Abstract

**Background:**

Non-motor symptoms (NMS) are important factors when selecting treatments for patients with advanced Parkinson’s disease (PD). We sought to elucidate the prescribing practices for advanced PD patients with NMS in Japanese clinical practice.

**Methods:**

We examined the prescription rates and doses of anti-PD drugs, and the use of non-steroidal anti-inflammatory drugs (NSAIDs) in post hoc analyses of a 52-week observational study of 996 PD patients with wearing-off on levodopa-containing therapy and ≥1 NMS.

**Results:**

Dopamine agonists were the most frequently prescribed drugs combined with levodopa-containing drugs, followed by entacapone, zonisamide, istradefylline, selegiline, and amantadine. The daily dose of levodopa-containing drugs, rotigotine, entacapone, istradefylline, and droxidopa, and the levodopa-equivalent dose increased during the observation period. In a subgroup analysis of patients stratified by NMS status (improved/unchanged/deteriorated), the deteriorated group had higher prescription rates of entacapone and istradefylline, whereas the improved group had higher prescription rates of NSAIDs and zonisamide at Week 52. Prescriptions varied by geographical region for anti-PD drugs and by NMS status for NSAIDs.

**Conclusions:**

There were significant changes in the prescriptions and dosing of selected anti-PD drugs, especially newer drugs. Anti-PD drug and NSAID prescriptions also varied by changes in NMS status and geographic region.

## Introduction

Patients with Parkinson’s disease (PD) often exhibit a range of motor symptoms, such as wearing-off and dyskinesia, that are primarily caused by chronic levodopa therapy and have a significant impact on the patient’s daily activities and quality of life [1, 2]. Many patients also develop non-motor symptoms (NMS), such as pain, cognitive symptoms, neuropsychiatric symptoms, sensory symptoms, sleep disorders, and autonomic symptoms, that also contribute to the deteriorations in quality of life [3–6]. Thus, it is important for physicians to consider not only the motor symptoms but also the NMS in the care of patients with PD.

The clinical guidelines for PD currently contain limited evidence regarding the use of medications to manage NMS and only describe medications based on experience [7]. Furthermore, there are limited data on the best practices for the pharmacological treatment of PD encompassing the treatment of NMS. Accordingly, there is a clear need to obtain more data, particularly in real-world settings, regarding the use of appropriate medications for NMS.

Some studies have attempted to analyze the real-world treatment of PD in Japan, including recent analyses of health insurance claims data using the Japanese Medical Data Vision database, for example [8, 9]. Although such studies provide a snapshot of recent treatment practices, they contained limited data on disease severity and NMS burden, which should influence treatment practices. Therefore, studies using prospectively collected data in clinical practice can help to investigate the therapies prescribed by physicians in the real world.

We should also consider the possibility that treatment practices may vary among clinical institutions and regions. Indeed, geographical variability in prescribing practices has been reported in other countries/continents that may reflect differences in national guidelines or an urban–rural divide [10–12]. In particular, in a European study, the use of anti-PD drugs (calculated as the defined daily dose per 1000 inhabitants daily) increased between 2003 and 2007, but there were clear differences in this measure, as well as the use of specific drug classes, among the countries evaluated in that study [12].

Some anti-PD drugs are used to treat the NMS of PD, but they can sometimes lead to the development or exacerbation of NMS, which can be difficult to manage. Another factor of interest is the use of non-steroidal anti-inflammatory drugs (NSAIDs) for managing pain in patients with PD. Recent studies have reported that NSAIDs may reduce the risk of PD in some individuals and could reduce CNS inflammation, which may be involved in some NMS [13–17].

Overall, more insight into the prescribing practices of anti-PD drugs and NSAIDs for PD in Japan is needed, including knowledge about changes in prescribing practices according to the progression of PD. Such data could be useful for optimizing the management of NMS to improve upon holistic approaches of care, which encompass all aspects of the life of PD patients.

The J-FIRST study, which started in 2014, was a 52-week observational study that was designed to gain a clearer picture surrounding NMS, motor symptoms, quality of life, and treatment practices in over 1000 Japanese patients with advanced PD across 35 study sites [18–21]. Of note, the study revealed marked differences in the patterns of NMS among Japanese patients, including sex-related differences in the prevalence and severity of NMS [18], as well as marked changes in NMS that were associated with changes in quality of life (assessed using the 8-item Parkinson’s Disease Questionnaire [PDQ-8]) over time [19]. Additionally, patient background characteristics were compared among patients divided into three groups based on the change in NMS (improved/unchanged/deteriorated) [19]. To better understand these earlier findings and to provide more insight into the management of Japanese patients with PD, we performed further analyses of the J-FIRST study to answer the following research questions:

1. Do the prescriptions of anti-PD drugs and NSAIDs change over time in patients with PD in Japan? To our knowledge, this is the first study to examine the prescription practices for NSAIDs in Japan.
2. How do the prescriptions for anti-PD drugs and NSAIDs change among Japanese patients stratified by whether their NMS improved, unchanged, or deteriorated based on their changes in Movement Disorder Society‒Unified Parkinson’s Disease Rating Scale (MDS-UPDRS) Part I scores?
3. Do the prescriptions of major anti-PD drugs and NSAIDs vary among regions of Japan?

## Methods

### Ethics

The study was approved by the Ethics Review Committees at all participating sites. The study was conducted in compliance with the Ethical Guidelines for Epidemiological Research in the spirit of the Declaration of Helsinki. All patients provided written informed consent prior to participation. The study was registered on ClinicalTrials.gov (NCT02073981) and the University Hospital Medical Information Network (UMIN) Clinical Trials Registry (umin.ac.jp/ctr/index-j.htm; UMIN000013161).

### Study design

The design of the study has been reported in more detail [18, 19]. Between March 1, 2014 and January 31, 2015, the study enrolled patients with advanced-stage PD aged ≥20 years who were displaying wearing-off on levodopa-containing therapy and had ≥1 NMS assessed using MDS-UPDRS Part I. This study was exploratory in nature, and it was challenging to plan the sample size from a confirmatory perspective. As we previously described [18], we set the sample size at 1000 patients considering the feasibility of the study and the required number of patients to identify factors related to NMS. Overall, 996 patients satisfied the eligibility criteria and were included in the present analyses as the overall sample. Patients were enrolled across 35 sites distributed throughout Japan. These sites were broadly divided into East and West Japan.

This was an observational study of clinical practice with a 52-week follow-up in which all treatments were prescribed at the attending physician’s discretion. At baseline, the physicians collected data regarding patient characteristics, comorbid motor symptoms and NMS, MDS-UPDRS Parts I and IV (4.3), modified Hoehn and Yahr stage (mH&Y, ON and OFF states), and PDQ-8. At Weeks 0, 13, 26, 39, and 52, the participating physicians also collected data regarding the prescriptions for anti-PD drugs and NSAIDs with their doses (**S1 Table**), together with MDS-UPDRS Part I scores. The MDS-UPDRS Part I scores at Weeks 0, 13, 26, 39, and 52 were used to categorize patients according to whether the NMS status of PD improved, unchanged, or deteriorated using the group-based trajectory models described in the previous article [19].

### Statistical analyses

In this study, we used the overall sample to determine the changes in prescription rates and doses over time for the anti-PD drugs and NSAIDs listed in **S1 Table**. These analyses were also repeated in patients divided into three groups according to the change in MDS-UPDRS Part I score (i.e., improved, unchanged, or deteriorated) [19] and in patients divided into two major geographical regions (East and West Japan). We used generalized linear models (GLM) for the analyses of prescription rates in the overall sample and in the patient subgroups (improved/unchanged/deteriorated and East/West Japan). The models of dosing in the overall sample were adjusted for age, sex, body mass index, duration of PD, age at PD onset, and mH&Y Score (ON). All these variables, except for body mass index, were used in the analysis by region. The results are presented as estimates ± standard error (SE). The estimates and SE for the prescription rates were originally calculated on a scale of 0–1 and converted into percentages on a scale of 0%–100% to aid interpretation. We determined *P*-values for the change from Week 0 to 52 in the analyses of the overall sample and for the analysis by the change in NMS status (i.e., improved/unchanged/deteriorated), but not for the analysis by region. All analyses were considered exploratory and *P*-values of <0.05 (two-sided) were considered significant without adjustment for multiple comparisons. SAS software (version 9.4; SAS Institute Inc., Cary, NC) was used for the analyses.

## Results

### Prescription status of the overall sample

As explained in a previous paper [19], 996 patients were enrolled and eligible for the analyses. There were 624 females (62.7%) and 372 males (37.3%), with a mean (standard deviation [SD]) age of 68.1 (8.8) years, mean (SD) PD duration of 10.9 (5.5) years, and mean (SD) age at PD onset of 58.1 (9.9) years. As expected from the eligibility criteria, the patients had advanced PD, with a mean (SD) number of comorbid NMS of 6.6 (2.5), a mean (SD) MDS-UPDRS Part I total score of 10.9 (5.4), and a mean (SD) MDS-UPDRS Part IV (4.3) score of 1.4 (0.7). According to the mH&Y scale, the ON state was classified as mild (0–2.5), moderate (3), and severe (4, 5) for 65.36%, 28.51%, and 5.92% of patients, respectively. The OFF state was classified as mild, moderate, and severe in 19.18%, 44.68%, and 35.84% of patients, respectively. The mean (SD) PDQ-8 total score at baseline was 7.3 (5.2). The mean (SD) dosage of levodopa-containing drugs was 436.4 (165.7) mg/day, and the mean (SD) levodopa-equivalent dose (LED) at baseline was 769.5 (339.0) mg/day.

**Fig 1:**
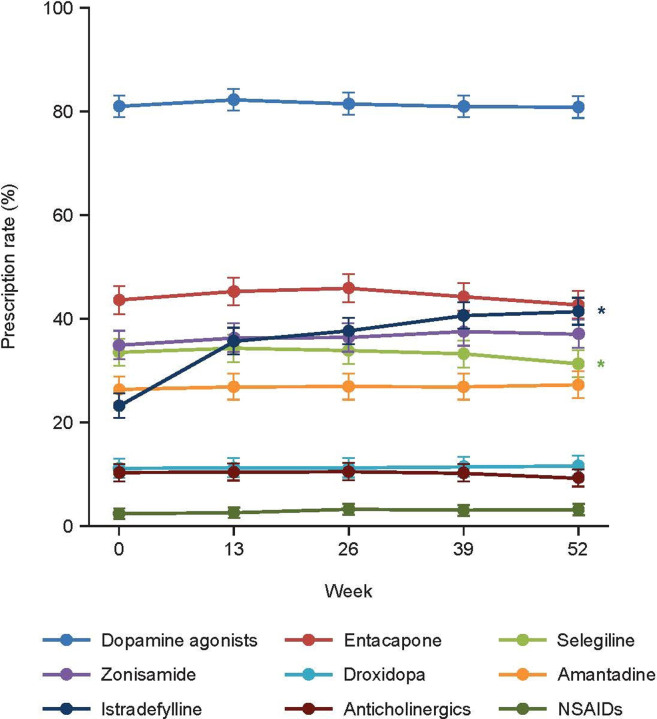
Prescription rates for antiparkinsonian drugs and non-steroidal anti-inflammatory drugs during the observation period for the overall sample. Values were calculated as estimates (0.0–1.0) ± standard error and converted to percentages (0%–100%). *P < 0.05 for Week 52 vs. Week 0 (generalized linear model). NSAIDs: non-steroidal anti-inflammatory drugs.

The anti-PD drugs and NSAIDs prescribed to patients in the overall sample during the 52-week observational period are shown in **Fig 1.** Dopamine agonists were the most frequently prescribed drugs (range: 80.8% to 82.2% of patients) used in combination with levodopa-containing drugs throughout the observation period. Other commonly used drugs were entacapone (42.6% to 45.9%), zonisamide (34.9% to 37.5%), istradefylline (23.2% to 41.4%), selegiline (31.3% to 34.3%), and amantadine (26.3% to 27.2%). The least frequently used drugs were droxidopa (11.1% to 11.6%), anticholinergics (9.2% to 10.5%), and NSAIDs (2.4% to 3.2%). The prescription rate for istradefylline increased significantly whereas the prescription rate for selegiline decreased significantly (both: *P* < 0.05, GLM) between Weeks 0 and 52 (**Fig 1**). Additionally, the prescription rate increased significantly for rotigotine and decreased significantly for pramipexole (both: *P* < 0.05, GLM) (**S1 Fig**).

**Fig 2:**
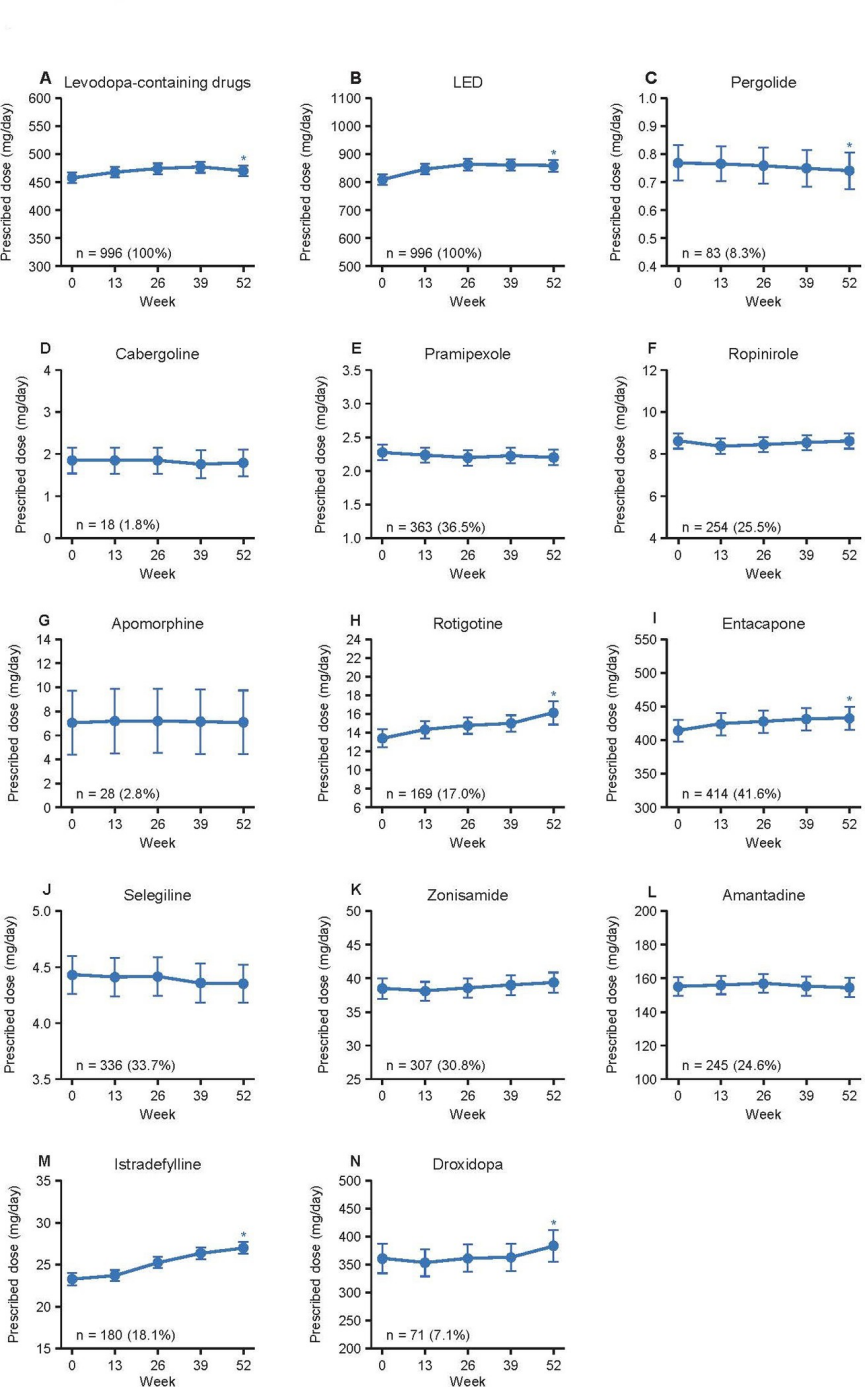
Prescribed doses of antiparkinsonian drugs during the observation period for the overall sample. (A) Levodopa-containing drugs. (B) Levodopa equivalent dose. (C) Pergolide. (D) Cabergoline. (E) Pramipexole. (F) Ropinirole. (G) Apomorphine. (H) Rotigotine. (I) Entacapone. (J) Selegiline. (K) Zonisamide. (L) Amantadine. (M) Istradefylline. (N) Droxidopa. Values are estimates ± standard error. The number of patients (%) prescribed each drug at Week 0 is indicated. *P < 0.05 for Week 52 vs. Week 0 (generalized linear model). LED: levodopa-equivalent dose.

Regarding the daily doses of anti-PD drugs, **Fig 2** shows there were significant increases from Week 0 to Week 52 in the doses of levodopa-containing drugs and the LED (both: *P* < 0.05, GLM). In particular, the dose of levodopa-containing drugs increased by 12.3 ± 5.8 mg/day (estimate ± SE) and LED increased by 49.0 ± 11.0 mg/day (estimate ± SE). Moreover, there were significant increases from Week 0 to Week 52 in the doses of rotigotine, entacapone, istradefylline and droxidopa, whereas there was a significant decrease in the dose of pergolide during this period (all: *P* < 0.05, GLM). The doses of other drugs remained stable.

### Prescription status according to the change in NMS status during the observation period

Patients were divided into three groups based on the pattern of MDS-UPDRS Part I scores during the 52-week observation period: improved (N = 161), unchanged (N = 635), and deteriorated (N = 200) [19]. The baseline characteristics of these groups are presented in **Table 1**. Although no formal statistical comparisons were made, some differences in the baseline characteristics were apparent. In particular, the improved group had higher baseline MDS-UPDRS Part I and more patients with severe mH&Y scores for ON and OFF states, indicative of more severe disease at baseline.

**Table 1:** Patient characteristics according to their change in non-motor symptoms during the observation period^a^.

	Improved	Unchanged	Deteriorated
N	161	635	200
Sex, n (%)			
Female	95 (59.01%)	404 (63.62%)	125 (62.50%)
Male	66 (40.99%)	231 (36.38%)	75 (37.50%)
Age (years)	67.7 (9.1)	68.4 (8.7)	67.4 (8.8)
PD duration (years)	11.0 (5.2)	11.1 (5.7)	10.4 (5.1)
BMI (kg/m^2^)	22.1 (3.1)	21.7 (3.5)	21.6 (3.8)
Age of PD onset (years)	57.7 (10.0)	58.3 (10.0)	58.1 (9.8)
Duration of comorbid motor symptoms (years)	9.2 (5.3)	8.9 (5.4)	8.2 (4.7)
Number of comorbid NMS	7.7 (2.2)	6.3 (2.4)	6.3 (2.6)
MDS-UPDRS Part I (total score)	15.4 (5.2)	10.3 (5.0)	9.1 (4.7)
MDS-UPDRS Part IV (4.3)	1.5 (0.7)	1.4 (0.7)	1.5 (0.7)
mH&Y (ON), n (%)			
Mild (0–2.5)	87 (54.04%)	428 (67.40%)	136 (68.00%)
Moderate (3)	54 (33.54%)	172 (27.09%)	58 (29.00%)
Severe (4, 5)	19 (11.80%)	34 (5.35%)	6 (3.00%)
Missing	1 (0.62%)	1 (0.16%)	0 (0.00%)
mH&Y (OFF), n (%)			
Mild (0–2.5)	27 (16.77%)	132 (20.79%)	32 (16.00%)
Moderate (3)	62 (38.51%)	278 (43.78%)	105 (52.50%)
Severe (4, 5)	72 (44.72%)	223 (35.12%)	62 (31.00%)
Missing	0 (0.00%)	2 (0.31%)	1 (0.50%)
PDQ-8 (total score)	9.1 (5.5)	6.8 (5.0)	7.3 (5.5)
Dose of levodopa-containing drugs (mg/day)	448.4 (191.1)	429.9 (158.3)	447.0 (166.2)
Levodopa-equivalent dose (mg/day)	816.3 (381.5)	746.5 (320.0)	804.7 (356.0)
Presence of dyskinesia, n (%)	77 (47.83%)	275 (43.31%)	98 (49.00%)

Values are means (SD) unless otherwise stated.

^a^Based on the change in MDS-UPDRS Part I total score [19].

BMI: body mass index; MDS-UPDRS: Movement Disorders Society-Unified Parkinson’s Disease Rating Scale; mH&Y: modified Hoehn and Yahr; NMS: non-motor symptoms; PD: Parkinson’s disease; PDQ-8: eight-item Parkinson’s Disease Questionnaire.

**Fig 3:**
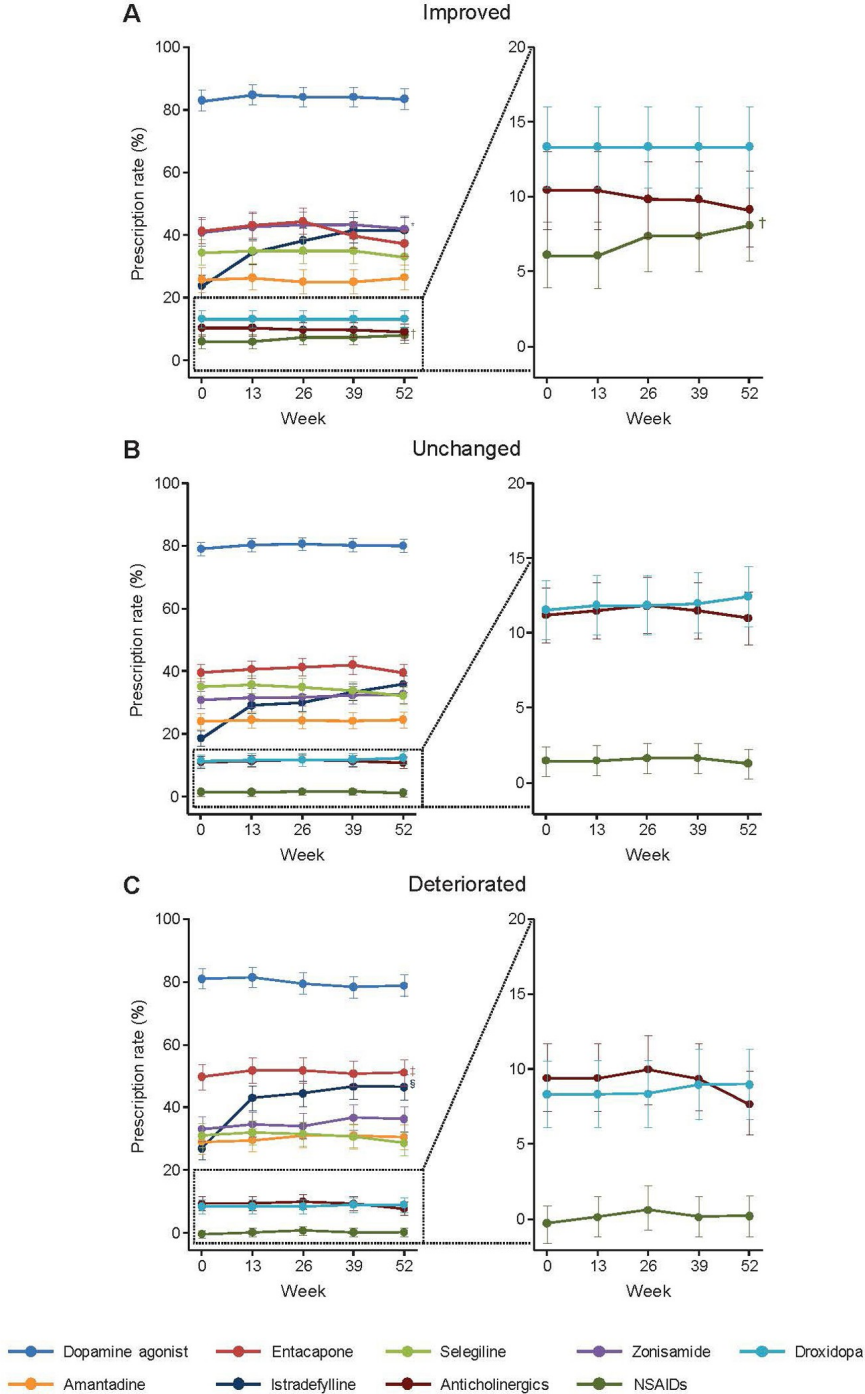
Prescription rates of antiparkinsonian drugs and non-steroidal anti-inflammatory drugs during the observation period in patients divided according to the pattern of change in non-motor symptoms: improved (A), unchanged (B), and deteriorated (C). Values are estimates ± standard error. Symbols indicate significance at a level of α = 0.05 (generalized linear model): *zonisamide—improved group vs. unchanged group; ^†^NSAIDs—improved group vs. unchanged and deteriorated groups; ^‡^entacapone—deteriorated group vs. improved and unchanged groups; ^§^istradefylline—deteriorated group vs. unchanged group. NSAIDs: non-steroidal anti-inflammatory drugs.

There were statistically significant differences in the prescription rates of several drugs at Week 52 among the three groups (**Fig 3**). The deteriorated group had a higher prescription rate of entacapone than the improved (*P* = 0.0079, GLM) and unchanged (*P* = 0.0040, GLM) groups, and had a higher prescription rate of istradefylline than the unchanged group (*P* = 0.0110, GLM). By comparison, the improved group had a higher prescription rate of zonisamide than the unchanged group (*P* = 0.0366, GLM). We also examined the change in the prescription rate of NSAIDs. At Week 52, the improved group had a higher prescription rate of NSAIDs than the unchanged (*P* = 0.0070, GLM) and deteriorated (*P* = 0.0031, GLM) groups (**Fig 3**).

**Fig 4:**
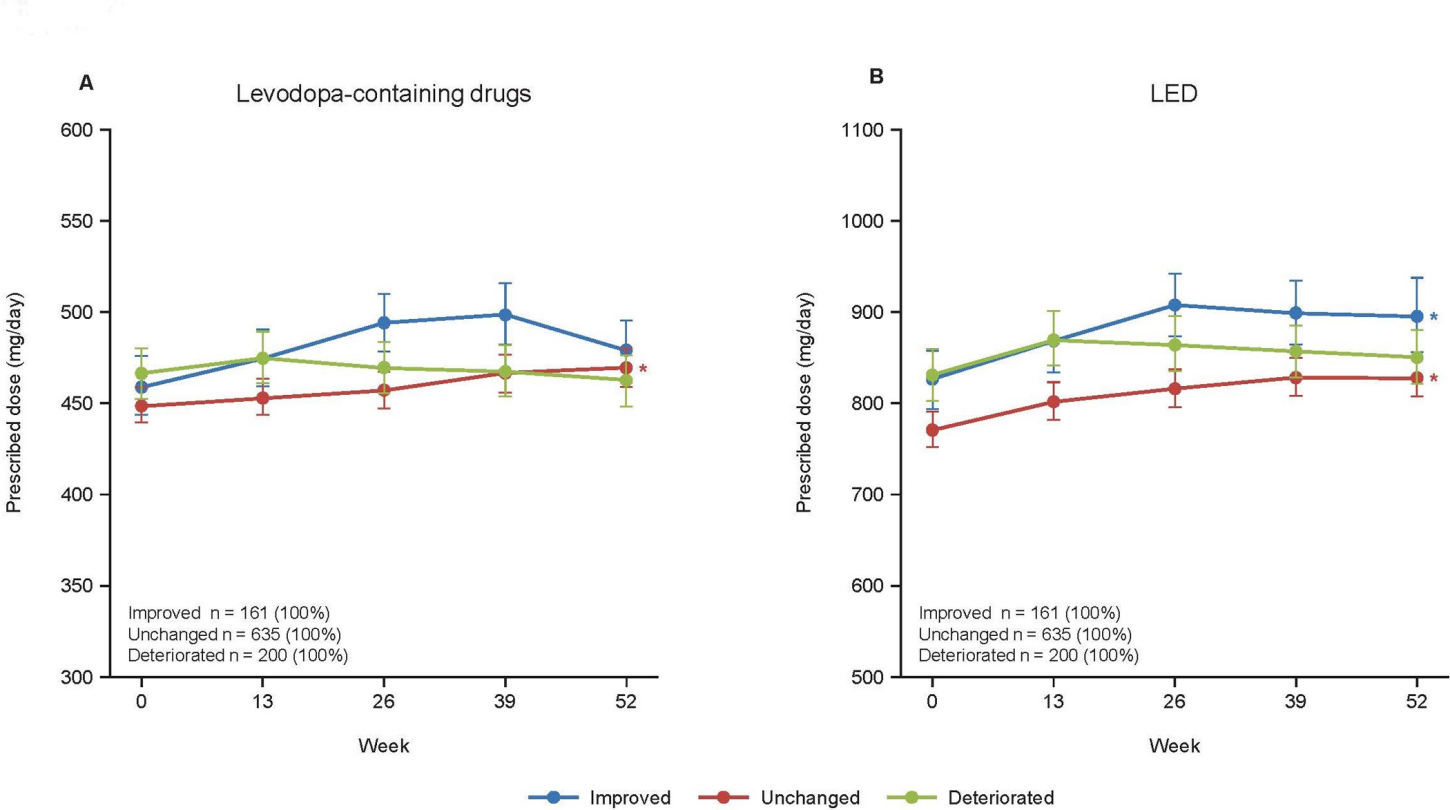
Prescribed doses of levodopa-containing drugs (A) and the levodopa-equivalent dose (B) during the observation period for the improved, unchanged, and deteriorated groups. Values are estimates ± standard error. The number of patients (%) in each group is indicated. *P < 0.05 for Week 52 vs. Week 0 (generalized linear model). LED: levodopa-equivalent dose.

Regarding the changes in daily doses of drugs, we found significant increases from Week 0 to Week 52 in the dose of levodopa-containing drugs in the unchanged group and the LED in the improved and unchanged groups (all *P* < 0.05, GLM; **Fig 4**), but there were no statistically significant differences among the three groups at Week 52. The improved group had a higher prescribed daily dose of entacapone at Week 52 than the unchanged (*P* = 0.0014) and deteriorated (*P* = 0.0437) groups (**S2 Fig**).

### Prescription status according to region

**Fig 5:**
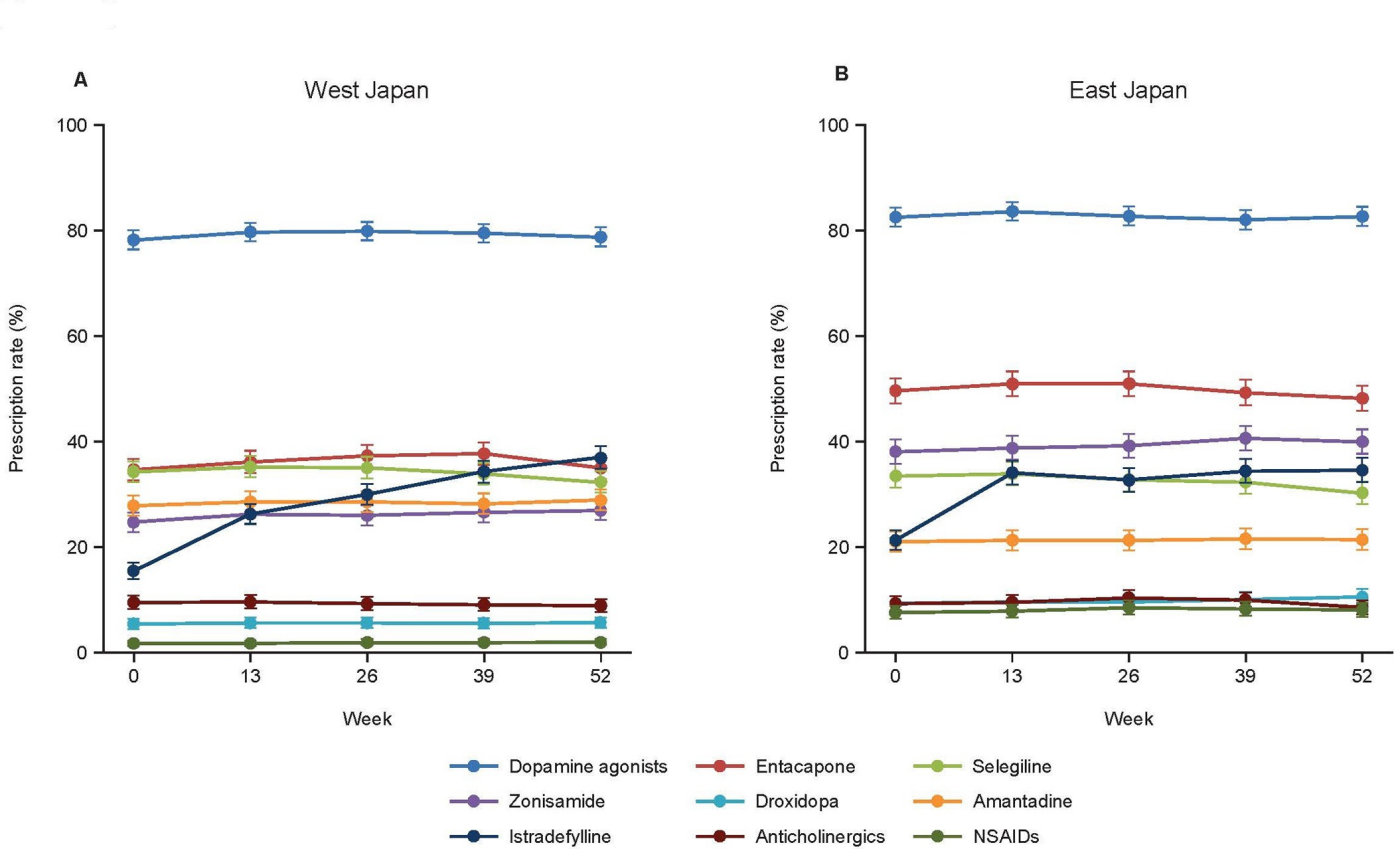
Prescription rates for antiparkinsonian drugs and non-steroidal anti-inflammatory drugs during the observation period for West (A) and East (B) Japan. Values were calculated as estimates (0.0–1.0) and converted to percentages (0%–100%). NSAIDs: non-steroidal anti-inflammatory drugs.

**Fig 6:**
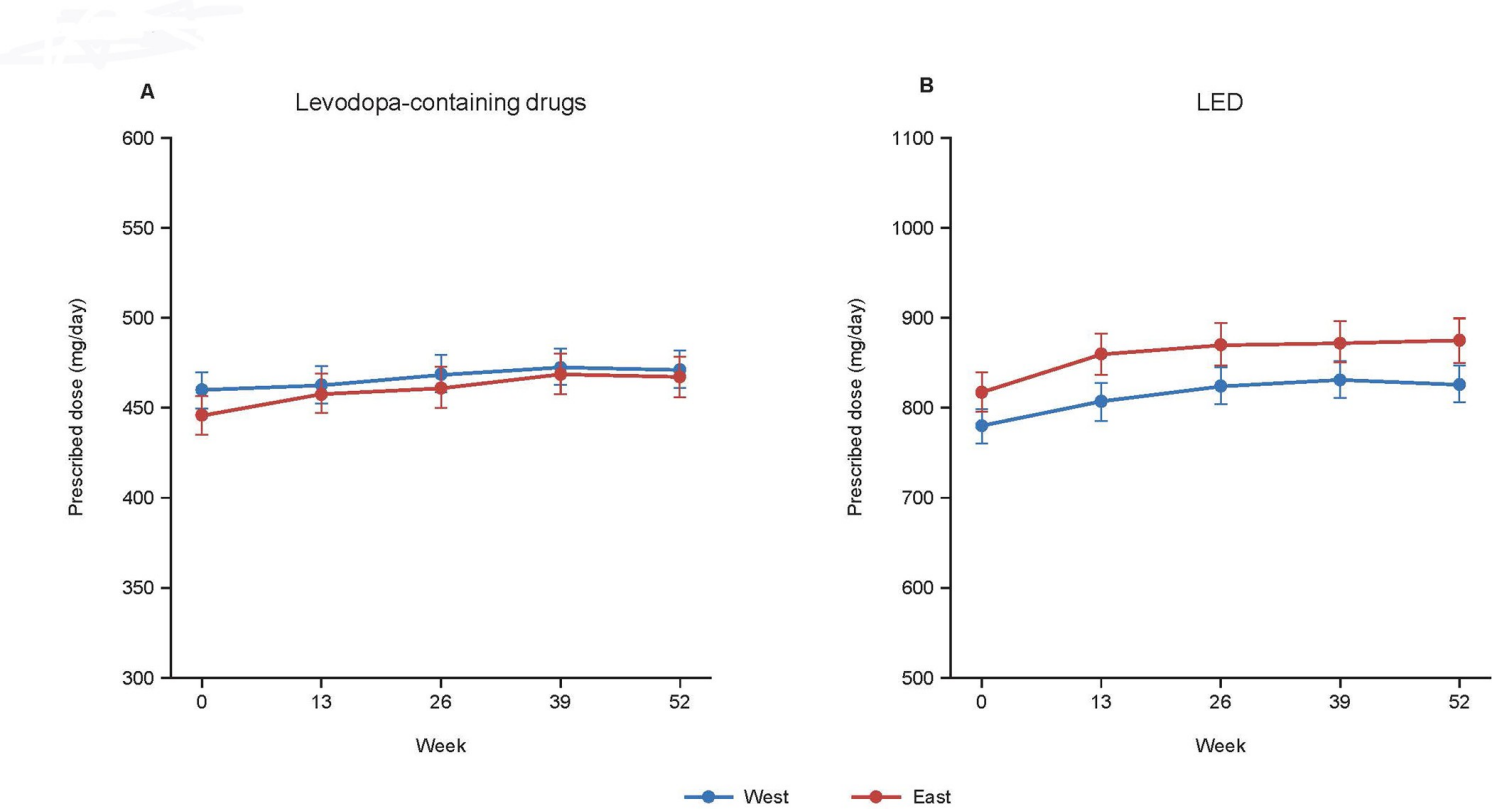
Prescribed doses of levodopa-containing drugs (A) and the levodopa-equivalent dose (B) during the observation period for West and East Japan. Values are estimates ± standard error. LED: levodopa-equivalent dose.

**Fig 5** depicts the prescription rates of anti-PD drugs and NSAIDs in West and East Japan separately. Some inconsistencies in the prescriptions of concomitant drugs between East and West Japan were apparent. Although there was no marked difference in the prescribed dose of levodopa-containing drugs between East and West Japan, the LED tended to be greater in East Japan (**Fig 6**).

## Discussion

We investigated the treatment practices for advanced PD patients who were taking levodopa-containing drugs and displaying wearing-off, and enrolled in the observational J-FIRST study across 35 sites between February 2014 and December 2016. In this cohort of patients taking levodopa-containing drugs, dopamine agonists were the predominant combination treatment throughout the observation period, being prescribed to 80.8% to 82.2% of patients, whereas entacapone was the second-most frequent drug, being prescribed to 42.6% to 45.9% of patients. In the overall sample, it can be inferred that the prescription rates and doses of newer anti-PD drugs (e.g., rotigotine and istradefylline) increased during the observation period, whereas the prescription rates decreased for older classes of anti-PD drugs (e.g., pramipexole and selegiline). Rotigotine and istradefylline were launched in Japan in 2013, just prior to the start of the J-FIRST study, and the increase in the prescription rates and doses may be due to the expectations for these drugs. Rotigotine was launched earlier (2006 in the EU and 2007 in the USA), and some reports have examined its efficacy for NMS [22, 23]. Another possible explanation is that because rotigotine is a transdermal patch, its use is increasing especially among patients with dysphagia who find it difficult to swallow oral drugs. Rotigotine may also be prescribed to patients with gastrointestinal disorders that interfere with drug absorption. Istradefylline is a non-dopaminergic drug, and expectations for its effects on NMS may have been high, and it may have been prescribed to patients whose NMS had not improved sufficiently with dopaminergic drugs. Over time, physicians may have become more aware of the properties and clinical efficacy of the newer anti-PD drugs, and hence they may have become more willing to prescribe them at appropriate doses in clinical practice.

Examining the prescription trends in clinical trials conducted in other countries of advanced PD patients who were also taking levodopa-containing drugs, the dosage of levodopa-containing drugs at baseline ranged from 572 to 821 mg/day in earlier studies [24–27], as compared with 436.4 mg/day in the J-FIRST study. Differences were observed in the prescription rates at baseline in other countries (dopamine agonists, 57.1%–91.3%; monoamine oxidase B [MAO-B] inhibitor, 10.5%–21.8%; catechol-*O*-methyl transferase [COMT] inhibitor, 23.3%–50.3%; amantadine, 13.0%–26.1%; anticholinergics, 9.5%–39.2%) as compared to the J-FIRST study (dopamine agonists, 80.9%; MAO-B inhibitor, 33.5%; COMT inhibitor, 43.6%; amantadine, 26.3%; anticholinergics, 10.3%). These values in J-FIRST are also very similar to those of other recent studies in Japan (dose of levodopa-containing drugs, 399.3–446.5 mg/day; dopamine agonists, 72.1%–95.7%; MAO-B inhibitor, 47.1%–55.5%; COMT inhibitor, 12.7%–45.6%; amantadine, 16.3%–38.1%; anticholinergics, 5.1%–19.5%) [28–31]. Although the data should not be directly compared among studies, the dose of levodopa-containing drugs tended to be lower in Japan than in other countries (as described above and in [32]), whereas the prescription rates of other drugs tended to be similar or slightly higher in Japan than in other countries. These differences in prescription trends suggest that Japanese physicians are attempting to control the symptoms of advanced PD by combining levodopa-containing drugs with other anti-PD drugs, without increasing the dose of levodopa-containing drugs.

In our study, the unchanged group showed a significant change in the dose of levodopa-containing drugs, and the improved and unchanged groups showed significant changes in LED. These changes may have helped prevent/delay disease progression and/or led to improvements in NMS in these patients, especially when coupled with the increases in the doses and prescription rate of other anti-PD drugs and NSAIDs. The improved or deteriorated groups showed a tendency for higher prescriptions of some anti-PD drugs than the other group(s). A reason for this may be that these drugs may have been added to existing therapy due to worsening symptoms during the observation period in the deteriorated group, or contributed to the improvement in symptoms in the improved group. However, there was no clear evidence as to whether the symptoms improved/deteriorated due to the addition of these drugs. Regarding NSAIDs, some pharmacoepidemiologic reports have suggested that they can suppress the onset or progression of PD [13–15]. An earlier clinical study examined the relationship between NMS and inflammatory cytokines, and the authors suggested that NSAIDs may have an effect on NMS [33]. Although this could not be investigated in the present study with a 52-week observation, the improved group was prescribed NSAIDs more frequently, which may have contributed to the improvement of NMS. Therefore, prescribing NSAIDs may be a good adjunct to anti-PD drugs, provided that they are used with appropriate consideration for the potential side effects of NSAIDs, such as peptic ulcers.

The LED tended to be greater in East Japan than in West Japan. A reason for this may be that the prescription rate of concomitant drugs, other than levodopa-containing drugs, differed between East and West Japan. Unfortunately, due to the nature of the study, the reasons for these inconsistencies cannot be ascertained from the available data. Further studies may be useful to help understand the factors underlying the geographical inconsistencies of anti-PD drug and NSAID prescriptions in Japan.

### Limitations

There are limitations of the J-FIRST study that should be mentioned, beyond those described in the prior articles [18–21]. In particular, it was not possible to assess whether the changes in treatments were done in order to improve the patient’s NMS status or whether they were a response to clinical factors (e.g., tolerability or as a consequence of changes in other therapies). Motor deterioration is often a cause of therapy adjustments in PD. However, because the UPDRS III score was not measured in this study, we could not examine the relationship between motor deterioration and therapy adjustments. Further, it was not possible to determine the factors that may contribute to the geographical differences in anti-PD drug and NSAID prescriptions between East and West Japan. Additionally, the patients were enrolled between March 2014 and January 2015, predating the approval of new MAO-B inhibitors and a new COMT inhibitor. Thus, the results may not reflect current treatment practices in Japan.

### Conclusions

In conclusion, the results of this study have revealed significant changes in the prescriptions and doses of selected anti-PD drugs and NSAIDs for advanced PD patients with ≥1 NMS in Japan. Japanese physicians tended to increase the number of prescriptions and doses of newer drugs during the observation period. We also observed differences in anti-PD drug and NSAID prescriptions among subgroups of patients divided by their NMS status (improved/unchanged/deteriorated) and geographic region. Further studies might be useful to better understand these findings and evaluate their clinical relevance.

## Supporting information

S1 File List of J-FIRST investigators. (DOCX)

S1 Table Drugs evaluated in this study. (PDF)

S1 Fig Prescription rates of rotigotine (A) and pramipexole (B) during the observation period for the overall sample. Values were calculated as estimates (0.0–1.0) ± standard error and converted to percentages (0%–100%). *P < 0.05 for Week 52 vs. Week 0 (generalized linear model).
(PDF)

S2 Fig Prescribed doses of antiparkinsonian drugs during the observation period for the improved, unchanged, and deteriorated groups of patients: (A) pergolide, (B) cabergoline, (C) pramipexole, (D) ropinirole, (E) apomorphine, (F) rotigotine, (G) entacapone, (H) selegiline, (I) zonisamide, (J) amantadine, (K) istradefylline, and (L) droxidopa. Values are estimates ± standard error. The number of patients (%) prescribed each drug at baseline is indicated. *P < 0.05 for the improved group vs. the unchanged and deteriorated groups at Week 52 (generalized linear model).
(PDF)

## References

[1] Eusebi P, Romoli M, Paoletti FP, Tambasco N, Calabresi P, Parnetti L. Risk factors of levodopa-induced dyskinesia in Parkinson’s disease: results from the PPMI cohort. NPJ Parkinsons Dis. 2018;4:33. doi: 10.1038/s41531-018-0069-x . 30480086 PMC6240081

[2] Freitas ME, Hess CW, Fox SH. Motor complications of dopaminergic medications in Parkinson’s disease. Semin Neurol. 2017;37(2):147–157. doi: 10.1055/s-0037-1602423 . 28511255 PMC5990008

[3] Marinus J, Zhu K, Marras C, Aarsland D, van Hilten JJ. Risk factors for non-motor symptoms in Parkinson’s disease. Lancet Neurol. 2018;17(6):559–568. doi: 10.1016/s1474-4422(18)30127-3 . 29699914

[4] Prakash KM, Nadkarni NV, Lye WK, Yong MH, Tan EK. The impact of non-motor symptoms on the quality of life of Parkinson’s disease patients: a longitudinal study. Eur J Neurol. 2016;23(5):854–860. doi: 10.1111/ene.12950 . 26806538

[5] Santos-García D, de Deus Fonticoba T, Suárez Castro E, Aneiros Díaz A, McAfee D, Catalán MJ, et al. Non-motor symptom burden is strongly correlated to motor complications in patients with Parkinson’s disease. Eur J Neurol. 2020;27(7):1210–1223. doi: 10.1111/ene.14221 . 32181979

[6] Schapira AHV, Chaudhuri KR, Jenner P. Non-motor features of Parkinson disease. Nat Rev Neurosci. 2017;18(7):435–450. doi: 10.1038/nrn.2017.62 . 28592904

[7] Japanese Society of Neurology, Parkinson’s disease clinical practice guidelines 2018. Available from: https://www.neurology-jp.org/guidelinem/parkinson_2018.html. Accessed December 3, 2022. In Japanese.

[8] Suzuki M, Arai M, Hayashi A, Ogino M. Prescription pattern of anti-Parkinson’s disease drugs in Japan based on a nationwide medical claims database. eNeurologicalSci. 2020;20:100257. doi: 10.1016/j.ensci.2020.100257 . 32775705 PMC7397691

[9] Suzuki M, Arai M, Hayashi A, Ogino M. Adherence to treatment guideline recommendations for Parkinson’s disease in Japan: a longitudinal analysis of a nationwide medical claims database between 2008 and 2016. PLoS One. 2020;15(4):e0230213. doi: 10.1371/journal.pone.0230213 . 32330133 PMC7182259

[10] Bruno MK, Watanabe G, Gao F, Seto T, Nakagawa K, Trinacty C, et al. Difference in rural and urban Medicare prescription pattern for Parkinson’s disease in Hawai’i. Clin Park Relat Disord. 2022;6:100144. doi: 10.1016/j.prdoa.2022.100144 . 35521293 PMC9062359

[11] Kalilani L, Friesen D, Boudiaf N, Asgharnejad M. The characteristics and treatment patterns of patients with Parkinson’s disease in the United States and United Kingdom: A retrospective cohort study. PLoS One. 2019;14(11):e0225723. doi: 10.1371/journal.pone.0225723 . 31756215 PMC6874315

[12] Rosa MM, Ferreira JJ, Coelho M, Freire R, Sampaio C. Prescribing patterns of antiparkinsonian agents in Europe. Mov Disord. 2010;25(8):1053–1060. doi: 10.1002/mds.23038 . 20222132

[13] Esposito E, Di Matteo V, Benigno A, Pierucci M, Crescimanno G, Di Giovanni G. Non-steroidal anti-inflammatory drugs in Parkinson’s disease. Exp Neurol. 2007;205(2):295–312. doi: 10.1016/j.expneurol.2007.02.008 . 17433296

[14] Fyfe I. Aspirin and ibuprofen could lower risk of LRRK2 Parkinson disease. Nat Rev Neurol. 2020;16(9):460. doi: 10.1038/s41582-020-0394-7 . 32719503

[15] San Luciano M, Tanner CM, Meng C, Marras C, Goldman SM, Lang AE, et al. Nonsteroidal anti-inflammatory use and LRRK2 Parkinson’s disease penetrance. Mov Disord. 2020;35(10):1755–1764. doi: 10.1002/mds.28189 . 32662532 PMC7572560

[16] Singh A, Tripathi P, Singh S. Neuroinflammatory responses in Parkinson’s disease: relevance of ibuprofen in therapeutics. Inflammopharmacology. 2021;29(1):5–14. doi: 10.1007/s10787-020-00764-w . 33052479

[17] Swiątkiewicz M, Zaremba M, Joniec I, Członkowski A, Kurkowska-Jastrzębska I. Potential neuroprotective effect of ibuprofen, insights from the mice model of Parkinson’s disease. Pharmacol Rep. 2013;65(5):1227–1236. doi: 10.1016/s1734-1140(13)71480-4 . 24399718

[18] Maeda T, Shimo Y, Chiu SW, Yamaguchi T, Kashihara K, Tsuboi Y, et al. Clinical manifestations of nonmotor symptoms in 1021 Japanese Parkinson’s disease patients from 35 medical centers. Parkinsonism Relat Disord. 2017;38:54–60. doi: 10.1016/j.parkreldis.2017.02.024 . 28279596

[19] Watanabe H, Saiki H, Chiu SW, Yamaguchi T, Kashihara K, Tsuboi Y, et al. Real-world nonmotor changes in patients with Parkinson’s disease and motor fluctuations: J-FIRST. Mov Disord Clin Pract. 2020;7(4):431–439. doi: 10.1002/mdc3.12939 . 32373660 PMC7197319

[20] Shimo Y, Maeda T, Chiu SW, Yamaguchi T, Kashihara K, Tsuboi Y, et al. Influence of istradefylline on non-motor symptoms of Parkinson’s disease: A subanalysis of a 1-year observational study in Japan (J-FIRST). Parkinsonism Relat Disord. 2021;91:115–120. doi: 10.1016/j.parkreldis.2021.09.015 . 34583302

[21] Mishima T, Chiu SW, Saiki H, Yamaguchi T, Shimo Y, Maeda T, et al. Risk factors for developing dyskinesia among Parkinson’s disease patients with wearing-off: J-FIRST. J Neurol Sci. 2023;448:120619. doi: 10.1016/j.jns.2023.120619 . 37023638

[22] Sanford M, Scott LJ. Rotigotine transdermal patch: a review of its use in the treatment of Parkinson’s disease. CNS Drugs. 2011;25(8):699–719. doi: 10.2165/11206750-000000000-00000 . 21790211

[23] Zesiewicz TA, Martinez-Martin P. Effects of rotigotine transdermal system on non-motor symptoms in Parkinson’s disease: an overview. Expert Rev Neurother. 2013;13(12):1329–1342. doi: 10.1586/14737175.2013.859986 . 24236902

[24] Parkinson Study Group. A randomized placebo-controlled trial of rasagiline in levodopa-treated patients with Parkinson disease and motor fluctuations: the PRESTO study. Arch Neurol. 2005;62(2):241–248. doi: 10.1001/archneur.62.2.241 . 15710852

[25] Borgohain R, Szasz J, Stanzione P, Meshram C, Bhatt MH, Chirilineau D, et al. Two-year, randomized, controlled study of safinamide as add-on to levodopa in mid to late Parkinson’s disease. Mov Disord. 2014;29(10):1273–1280. doi: 10.1002/mds.25961 . 25044402

[26] Lees AJ, Ferreira J, Rascol O, Poewe W, Rocha JF, McCrory M, et al. Opicapone as adjunct to levodopa therapy in patients with Parkinson disease and motor fluctuations: a randomized clinical trial. JAMA Neurol. 2017;74(2):197–206. doi: 10.1001/jamaneurol.2016.4703 . 28027332

[27] Pourcher E, Fernandez HH, Stacy M, Mori A, Ballerini R, Chaikin P. Istradefylline for Parkinson’s disease patients experiencing motor fluctuations: results of the KW-6002-US-018 study. Parkinsonism Relat Disord. 2012;18(2):178–184. doi: 10.1016/j.parkreldis.2011.09.023 . 22000279

[28] Hattori N, Takeda A, Takeda S, Nishimura A, Kato M, Mochizuki H, et al. Efficacy and safety of adjunctive rasagiline in Japanese Parkinson’s disease patients with wearing-off phenomena: a phase 2/3, randomized, double-blind, placebo-controlled, multicenter study. Parkinsonism Relat Disord. 2018;53:21–27. doi: 10.1016/j.parkreldis.2018.04.025 . 29748109

[29] Hattori N, Tsuboi Y, Yamamoto A, Sasagawa Y, Nomoto M. Efficacy and safety of safinamide as an add-on therapy to L-DOPA for patients with Parkinson’s disease: a randomized, double-blind, placebo-controlled, phase II/III study. Parkinsonism Relat Disord. 2020;75:17–23. doi: 10.1016/j.parkreldis.2020.04.012 . 32446176

[30] Mizuno Y, Hasegawa K, Kondo T, Kuno S, Yamamoto M. Clinical efficacy of istradefylline (KW-6002) in Parkinson’s disease: a randomized, controlled study. Mov Disord. 2010;25(10):1437–1443. doi: 10.1002/mds.23107 . 20629136

[31] Takeda A, Takahashi R, Tsuboi Y, Nomoto M, Maeda T, Nishimura A, et al. Randomized, controlled study of opicapone in Japanese parkinson’s patients with motor fluctuations. Mov Disord. 2021;36(2):415–423. doi: 10.1002/mds.28322 . 33073879 PMC7983910

[32] Nagai M. International differences in approved and used dosing for drugs. Jpn J Clin Pharmacol Ther. 2011;42(2):59–60. doi: 10.3999/jscpt.42.59 (in Japanese; Table 3 in English).

[33] Lindqvist D, Kaufman E, Brundin L, Hall S, Surova Y, Hansson O. Non-motor symptoms in patients with Parkinson’s disease—correlations with inflammatory cytokines in serum. PLoS One. 2012;7(10):e47387. doi: 10.1371/journal.pone.0047387 23082161 PMC3474801

