# Supplementary material for: Prescription trends in Japanese advanced Parkinson’s disease patients with non-motor symptoms: J-FIRST
Source: PLoS One. 2024 Oct 23;19(10):e0309297. doi: 10.1371/journal.pone.0309297 (PMC11498663; doi:10.1371/journal.pone.0309297)
Supplement: S1 File — (DOCX) [file pone.0309297.s001.docx]

# **Supporting Information: J-FIRST study investigators**

Takashi Abe (Abe Neurology Clinic); Shih-Wei Chiu (Tohoku University Graduate School of Medicine); Kazuko Hasegawa (National Hospital Organization Sagamihara National Hospital); Nobutaka Hattori (Juntendo University Hospital); Tatsuya Hattori (Honmachi Clinic); Takanori Hazama (Osaka General Medical Center); Shigeki Hirano (Chiba University Graduate School of Medicine); Koichi Hirata (Dokkyo Medical University); Mutsumi Iijima (Tokyo Women’s Medical University School of Medicine); Mizuki Ito (Nagoya University Graduate School of Medicine); Kazunori Itoh (Iwamizawa Neurology Clinic); Satoshi Kamei (Nihon University School of Medicine); Kenichi Kashihara (Okayama Kyokuto Hospital); Ryoichi Kurisaki (National Hospital Organization Kumamoto-Minami National Hospital); Hirofumi Kusaka (Kansai Medical University); Seiji Kikuchi (National Hospital Organization Hokkaido Medical Center); Tetsuya Maeda (Iwate Medical University, Research Institute for Brain and Blood Vessels-Akita); Masahito Mihara (Osaka University Graduate School of Medicine); Hideto Miwa (Juntendo University Nerima Hospital); Takafumi Miyachi (Yanai Medical Center); Hideo Mori (Juntendo University Koshigaya Hospital); Miho Murata (National Center of Neurology and Psychiatry); Hiroshi Nagayama (Nippon Medical School Graduate School of Medicine); Kazutoshi Nishiyama (Kitasato University School of Medicine); Masahiro Nomoto (Ehime University Hospital); Ryuichi Ohkubo (Fujimoto General Hospital); Ryuji Saigo (Fujimoto General Hospital); Hidemoto Saiki (Kitano Hospital); Yasushi Shimo (Juntendo University Hospital); Atsushi Takeda (National Hospital Organization Sendai-Nishitaga Hospital); Makio Takahashi (Osaka Red Cross Hospital); Ryosuke Takahashi (Kyoto University Graduate School of Medicine); Yoshihisa Tatsuoka (Tatsuoka Neurology Clinic); Kazuo Toda (Toda Internal Medicine & Rehabilitation Clinic); Masahiko Tomiyama (Aomori Prefectural Central Hospital); Yoshio Tsuboi (Fukuoka University Hospital); Hirohisa Watanabe (Nagoya University); Takuhiro Yamaguchi (Tohoku University Graduate School of Medicine); Mitsutoshi Yamamoto (Takamatsu Neurology Clinic); Junji Yoshinaga (Yoshinaga Neurology Clinic). Their roles and responsibilities are described in Watanabe et al. Mov. Disord. Clin. Pract. 7 (2020) 431–439.
