# Supplementary material for: Prescription trends in Japanese advanced Parkinson’s disease patients with non-motor symptoms: J-FIRST
Source: PLoS One. 2024 Oct 23;19(10):e0309297. doi: 10.1371/journal.pone.0309297 (PMC11498663; doi:10.1371/journal.pone.0309297)
Supplement: S1 Table — (PDF) [file pone.0309297.s002.pdf]

S1 Table. Drugs evaluated in this study.

Class	Drug names
Levodopa-containing drugs	Levodopa, levodopa-benserazide, levodopa-carbidopa, and levodopa-carbidopa -entacapone
Dopamine agonists	Bromocriptine, pergolide, cabergoline, talipexole, pramipexole, ropinirole, apomorphine, and rotigotine
COMT inhibitor	Entacapone
MAO-B inhibitor	Selegiline
Levodopa enhancer	Zonisamide
Noradrenaline replacement	Droxidopa
Dopamine release enhancer	Amantadine
Adenosine A_{2A} receptor antagonist	Istradefylline
Anticholinergics	Biperiden, profenamine, trihexyphenidyl, and piroheptine
NSAIDs	Indomethacin, etodolac, ketoprofen, methyl salicylate-glycyrrhetinic acid combination drug, diclofenac, celecoxib, felbinac, flurbiprofen, meloxicam, loxoprofen, and lornoxicam

COMT, catechol-*O*-methyltransferase; MAO-B, monoamine oxidase type B; NSAIDs, nonsteroidal anti-inflammatory drugs
