# Supplementary material for: Prescription trends in Japanese advanced Parkinson’s disease patients with non-motor symptoms: J-FIRST
Source: PLoS One. 2024 Oct 23;19(10):e0309297. doi: 10.1371/journal.pone.0309297 (PMC11498663; doi:10.1371/journal.pone.0309297)

S1 Fig. Prescription rates of rotigotine (A) and pramipexole (B) during the observation period for the overall sample. Values were calculated as estimates (0.0–1.0) ± standard error and converted to percentages (0%–100%). **P* < 0.05 for Week 52 vs. Week 0 (generalized linear model).

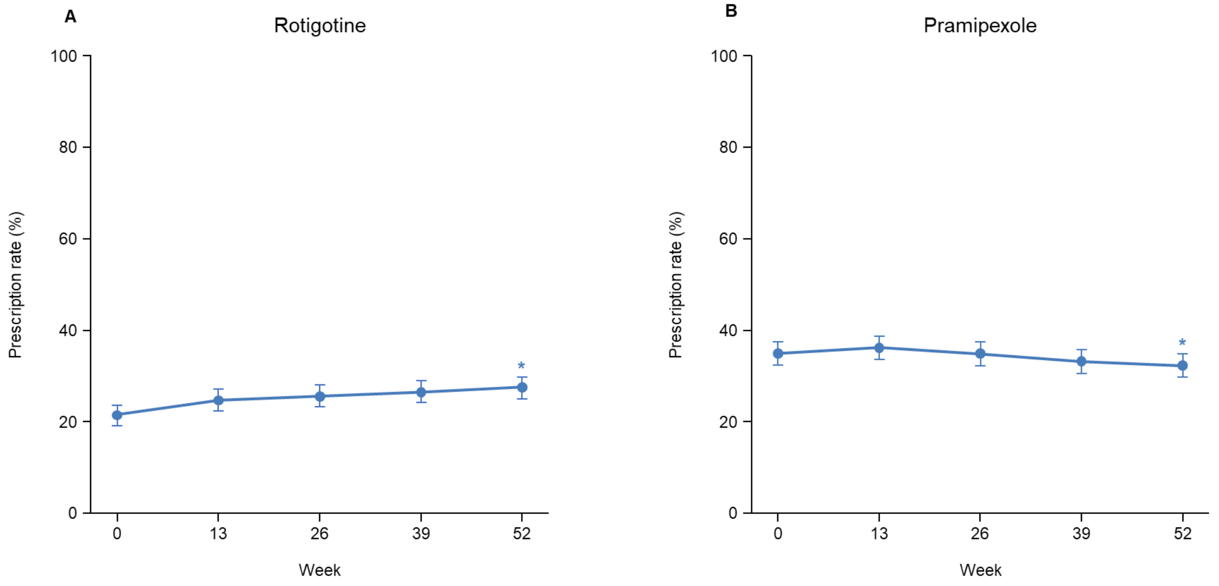

Supplement: S1 Fig — Prescription rates of rotigotine (A) and pramipexole (B) during the observation period for the overall sample. Values were calculated as estimates (0.0–1.0) ± standard error and converted to percentages (0%–100%). *P < 0.05 for Week 52 vs. Week 0 (generalized linear model). (PDF) [file pone.0309297.s003.pdf]
