# Supplementary material for: Prescription trends in Japanese advanced Parkinson’s disease patients with non-motor symptoms: J-FIRST
Source: PLoS One. 2024 Oct 23;19(10):e0309297. doi: 10.1371/journal.pone.0309297 (PMC11498663; doi:10.1371/journal.pone.0309297)
Supplement: S2 Fig — Prescribed doses of antiparkinsonian drugs during the observation period for the improved, unchanged, and deteriorated groups of patients: (A) pergolide, (B) cabergoline, (C) pramipexole, (D) ropinirole, (E) apomorphine, (F) rotigotine, (G) entacapone, (H) selegiline, (I) zonisamide, (J) amantadine, (K) istradefylline, and (L) droxidopa. Values are estimates ± standard error. The number of patients (%) prescribed each drug at baseline is indicated. *P < 0.05 for the improved group vs. the unchanged and deteriorated groups at Week 52 (generalized linear model). (PDF) [file pone.0309297.s004.pdf]

S2 Fig. Prescribed doses of antiparkinsonian drugs during the observation period for the improved, unchanged, and deteriorated groups of patients: (A) pergolide, (B) cabergoline, (C) pramipexole, (D) ropinirole, (E) apomorphine, (F) rotigotine, (G) entacapone, (H) selegiline, (I) zonisamide, (J) amantadine, (K) istradefylline, and (L) droxidopa. Values are estimates ± standard error. The number of patients (%) prescribed each drug at baseline is indicated. *$P < 0.05$ for the improved group vs. the unchanged and deteriorated groups at Week 52 (generalized linear model).

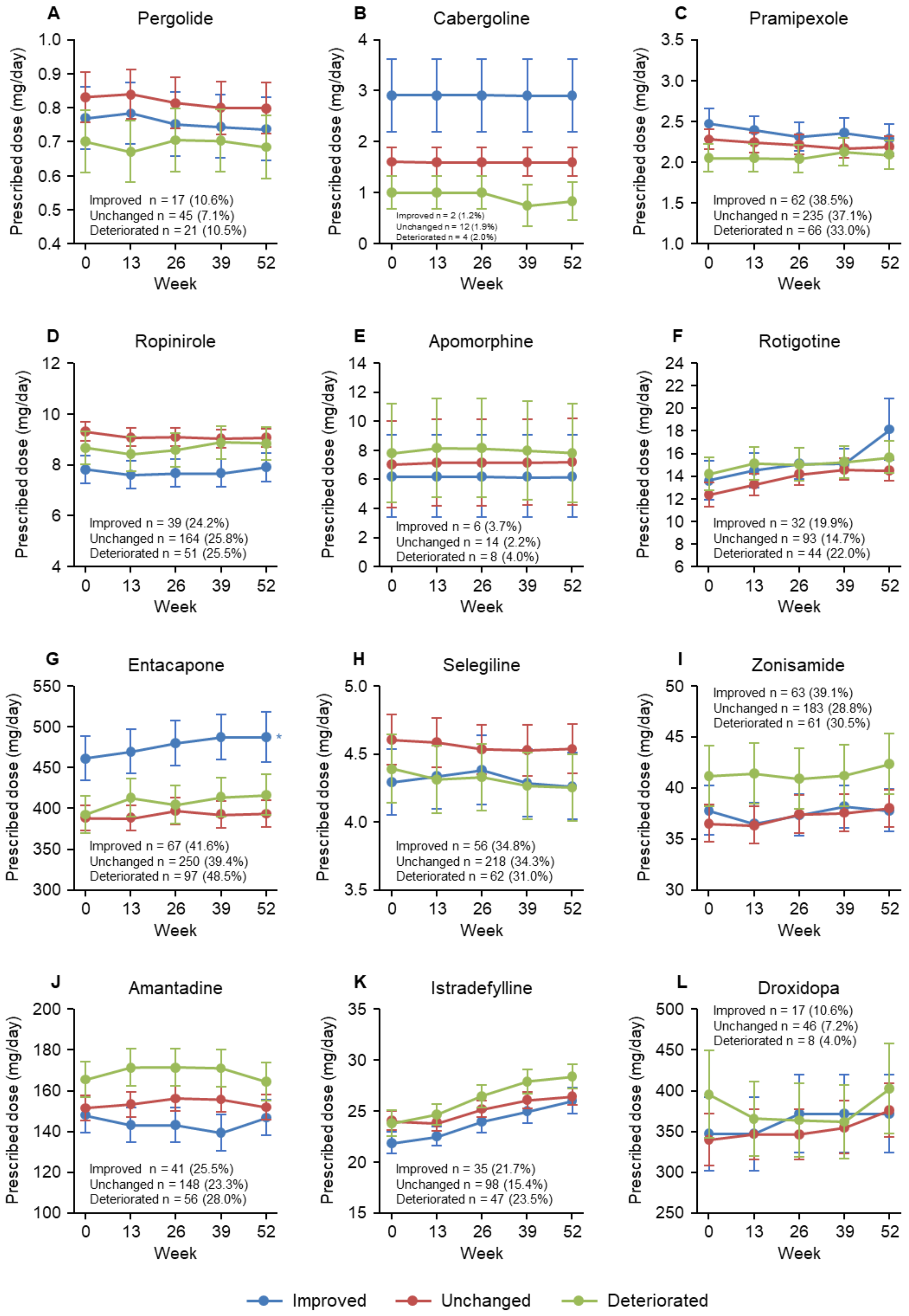
A
Pergolide
Improved n = 17 (10.6%)
Unchanged n = 45 (7.1%)
Deteriorated n = 21 (10.5%)
B
Cabergoline
Improved n = 2 (1.2%)
Unchanged n = 12 (1.9%)
Deteriorated n = 4 (2.0%)
C
Pramipexole
Improved n = 62 (38.5%)
Unchanged n = 235 (37.1%)
Deteriorated n = 66 (33.0%)
D
Ropinirole
Improved n = 39 (24.2%)
Unchanged n = 164 (25.8%)
Deteriorated n = 51 (25.5%)
E
Apomorphine
Improved n = 6 (3.7%)
Unchanged n = 14 (2.2%)
Deteriorated n = 8 (4.0%)
F
Rotigotine
Improved n = 32 (19.9%)
Unchanged n = 93 (14.7%)
Deteriorated n = 44 (22.0%)
G
Entacapone
Improved n = 67 (41.6%)
Unchanged n = 250 (39.4%)
Deteriorated n = 97 (48.5%)
H
Selegiline
Improved n = 56 (34.8%)
Unchanged n = 218 (34.3%)
Deteriorated n = 62 (31.0%)
I
Zonisamide
Improved n = 63 (39.1%)
Unchanged n = 183 (28.8%)
Deteriorated n = 61 (30.5%)
J
Amantadine
Improved n = 41 (25.5%)
Unchanged n = 148 (23.3%)
Deteriorated n = 56 (28.0%)
K
Istradefylline
Improved n = 35 (21.7%)
Unchanged n = 98 (15.4%)
Deteriorated n = 47 (23.5%)
L
Droxidopa
Improved n = 17 (10.6%)
Unchanged n = 46 (7.2%)
Deteriorated n = 8 (4.0%)
Prescribed dose (mg/day)
Week
Improved
Unchanged
Deteriorated
